# Automatic measurement of the Cobb angle for adolescent idiopathic scoliosis using convolutional neural network

**DOI:** 10.1038/s41598-023-41821-y

**Published:** 2023-09-04

**Authors:** Yoshihiro Maeda, Takeo Nagura, Masaya Nakamura, Kota Watanabe

**Affiliations:** Department of Orthopedic Surgery, Keio University School of Medicine, Tokyo, Japan

**Keywords:** Diseases, Medical research

## Abstract

This study proposes a convolutional neural network method for automatic vertebrae detection and Cobb angle (CA) measurement on X-ray images for scoliosis. 1021 full-length X-ray images of the whole spine of patients with adolescent idiopathic scoliosis (AIS) were used for training and segmentation. The proposed AI algorithm's results were compared with those of the manual method by six doctors using the intraclass correlation coefficient (ICC). The ICCs recorded by six doctors and AI were excellent or good, with a value of 0.973 for the major curve in the standing position. The mean error between AI and doctors was not affected by the angle size, with AI tending to measure 1.7°–2.2° smaller than that measured by the doctors. The proposed method showed a high correlation with the doctors’ measurements, regardless of the CA size, doctors’ experience, and patient posture. The proposed method showed excellent reliability, indicating that it is a promising automated method for measuring CA in patients with AIS.

## Introduction

Scoliosis is a structural abnormality of the spine that involves bending and rotating. Children aged 10–17 years who have scoliosis of unknown cause are classified as having adolescent idiopathic scoliosis (AIS)^1^, which is the most common type of scoliosis that occurs in children at puberty onset^2^. Standing whole-spine radiography is the standard imaging technique for evaluating the severity and characteristics of scoliosis^3^. Scoliosis treatment is typically based on the severity of the spinal deformity, which is indicated by the Cobb angle (CA), and the risk of progression, which is determined by bone maturity^4^. The CA is commonly used to quantify scoliosis severity^4^ and is measured by estimating the angle between the two lines of the vertebral endplates at the upper and lower ends of the curve^5^.

Until recently, manual spinal curvature measurements were made directly on X-ray images using a protractor; however, this technique is time-consuming and susceptible to relatively high interobserver and intraobserver variabilities^6^. The measurement error was reported to be approximately 3°–10°^6–10^, with measurements differing up to 5° even with the same end vertebrae^7,11^. The causes of measurement variability or errors are multifactorial and include improper selection of one or both end vertebrae and incorrect drawing of the lines through the endplates. Other potential causes of errors include the level of clinical experience of examiners and the magnitude of the curve, which may be a greater issue with larger curves^10^.

Semiautomatic CA evaluation became possible with the advent of digitization in computed radiography. The picture archiving and communications system (PACS) has a built-in feature that allows users to digitally draw the required vertebral line, and the system automatically measures the CA. This method is more reliable and less variable than manual measurement on printed X-ray images using a protractor^12,13^, and the measurement accuracy has improved to within ± 3.3° of the true value^14–21^. However, PACS requires manual selection of the appropriate end vertebrae by surgeons.

At present, the development of a fully automated software tool that can eliminate the problem of repeatability and improve the accuracy of CA measurements has gained interest. In recent years, deep convolutional neural networks (CNNs) have shown great potential in the field of medical image analysis^19,22^. Several studies have been conducted on the automatic measurement of the CA^15,18,23^. Unlike traditional machine-learning methods^24–28^, deep neural networks are superior in terms of feature extraction and can be trained for object detection and semantic segmentation^23^.

Machine learning uses algorithms to analyze data and make informed decisions based on what is learned from that data. Deep learning uses a hierarchical structure of algorithms to build an artificial neural network that is capable of learning and making intelligent decisions on its own^29^. End-to-end deep learning replaces a machine-learning system that requires multiple stages of processing from the input of data to the output of results via a single large neural network with multiple layers and modules that perform various processes. Thus, CNNs are a suitable choice for extracting vertebral regions. Recent successes in precise image segmentation have been achieved using a U-Net architecture^30^.

Wu et al. used the multiview correlation network (MVC-Net) for spine curvature estimation from multiview (anterior–posterior [AP] and lateral [LAT]) X-ray images to measure the CA^18^. However, their method required a biplanar imaging approach. Zhang et al. developed a computer-aided method using deep neural networks, but it still required manual intervention, such as vertebral patch assignment, and the mean absolute difference values in radiographs exceeded 5°, with intraclass correlation coefficients (ICCs) of 0.771–0.835, making it unreliable for measuring the CA^19^. Horng et al. proposed an automatic system based on the Residual U-Net for measuring the spinal curvature using AP view radiographs, which showed improved segmentation results over existing CNN methods. The ICC was > 0.93, which was better than that obtained via manual measurement, but only major curves could be measured^16,23^.

In the present study, we proposed a CNN method for automatic vertebrae detection and CA measurement on X-ray images. Our method uses simple black-and-white inverted X-ray images, including supine side-bending X-ray images, to evaluate the flexibility of curves, and X-rays taken while wearing a brace. Those radiographic conditions have not been tested in previous studies. In addition, our method could measure all curves, including minor and major curves.

## Materials and methods

### Overview

For this study, we used 1021 full-length X-ray images of the whole spine of patients with AIS taken at Keio University Hospital between 2009 and 2020 as training data. Medical ethical permission was obtained by the Ethics Committee of Keio University School of Medicine. Since this is a form of secondary use of previously obtained clinical data, informed consent was not required and was handled on an opt-out basis. All procedures performed in this study were in accordance with the ethical standards of the national research committee. Each X-ray image depicted a complete spine, including 12 thoracic vertebrae and 5 lumbar vertebrae, and was used for subsequent training and segmentation. The X-ray images were retrieved from the institution PACS, anonymized, and exported as an image. The radiographs used were retrieved from patients’ medical records. No additional radiographs were generated for the purpose of this study. The inclusion criterion was patients diagnosed with AIS, whereas the exclusion criteria were patients who had (1) other musculoskeletal or neurological disorders or congenital vertebral anomalies or (2) previous spinal surgery. X-ray images taken in standing, supine, supine side-bending, and wearing-brace positions at our hospital were used for training. A total of 106 images were used for testing and 155 images were used for evaluation. The radiographs included 40 standing, 40 supine, 52 supine side-bending, and 23 wearing-brace images. Because of its clinical significance, we evaluated the CA on supine side-bending images in the direction that corrects the curves by supine side-bending.

The results of our proposed artificial intelligence (AI) algorithm were compared with those of the manual method using ZedView (LEXI Co., Ltd., Tokyo, Japan) by six doctors with different levels of experience (two experts who specialize in scoliosis treatment, two intermediates who were spine specialists, and two novices who were doctors in their third year of post-graduate studies). Proximal thoracic, main thoracic, and thoracolumbar/lumbar curves were evaluated, which were classified as major, minor 1, and minor 2 curves in the order of the magnitude of the CA.

**Figure 1:**
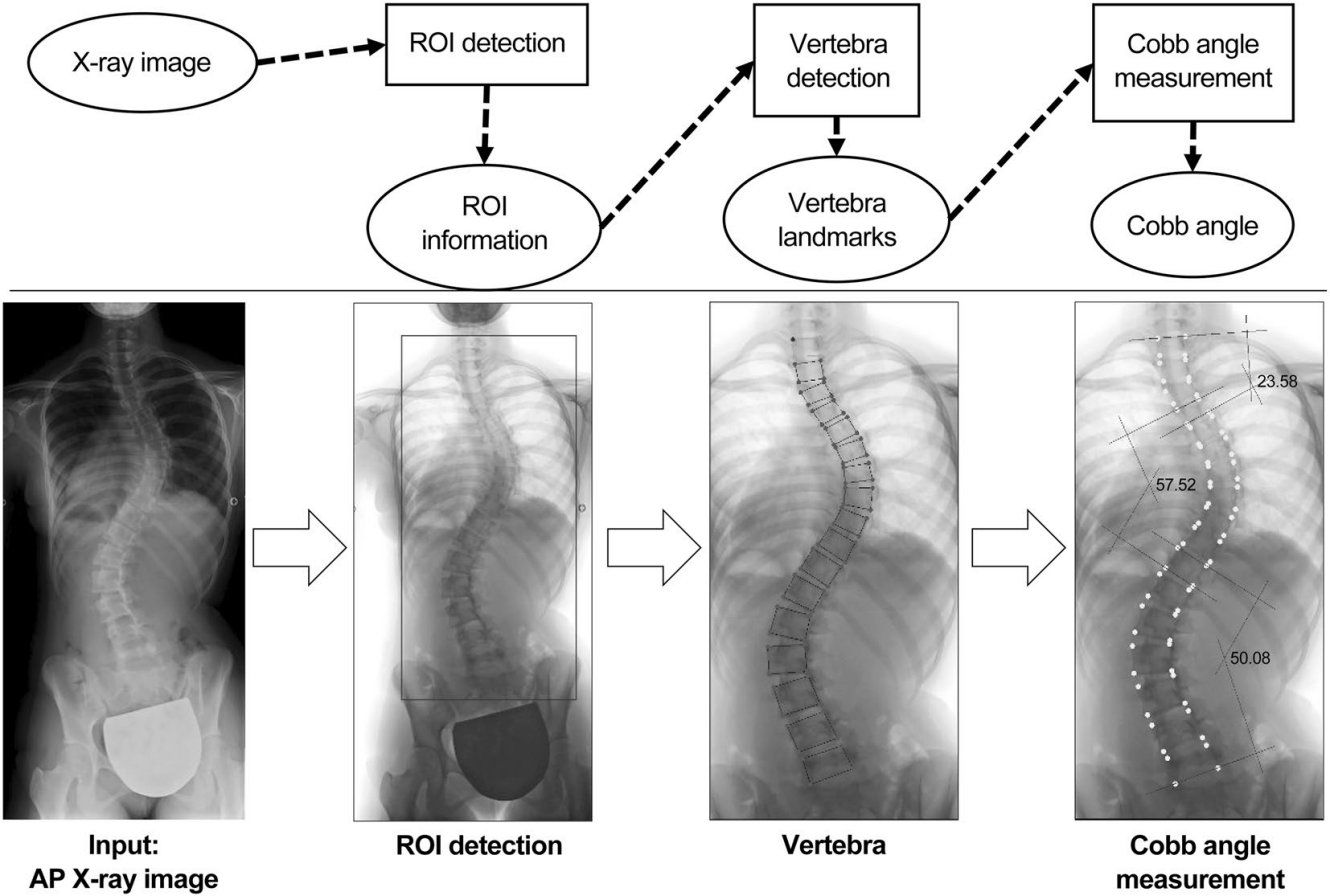
Schematic of the proposed algorithm. In the first stage, a region of interest (ROI) is identified on the X-ray image, which includes the whole spine with 12 thoracic and 5 lumbar vertebrae. In the second stage, the four corners of each vertebra are detected as feature points for the 17 vertebral bodies from T1 to L5 in the ROI. In the final stage, the detected feature points are used to measure the major and minor curves of the CA.

Our proposed algorithm consists of three stages (Fig. 1). In the first stage, a region of interest (ROI) is identified on the X-ray image, which includes the whole spine with 12 thoracic and 5 lumbar vertebrae. In the second stage, the four corners of each vertebra are detected as feature points for the 17 vertebral bodies from T1 to L5 in the ROI. In the final stage, the detected feature points are used to measure the major and minor curves of the CA.

For the ROI detection stage and vertebra detection stage, we performed transfer learning based on the pretrained model of Residual Network (ResNet)^31^, a method known for its superior performance in image recognition tasks in machine learning.

### ROI detection

The purpose of the ROI detection step is to identify the region of spinal deformity in a given X-ray image. In this step, the XY coordinate values of the upper-left and lower-right corners of the rectangle indicating the region of spinal deformity are detected in the X-ray image. As the network architecture, we performed transfer learning based on the pretrained model of ResNet34^31^. The input size of the network was 512 × 512 × 3, and a gray-scale image of size 512 × 512 was used as the input. Transfer learning was performed by replacing the output layer of ResNet34 with a four-channel fully connected layer. The network output was trained with four real values from 0 to 1 representing the XY coordinates of the upper-left and lower-right corners of the ROI in the thoracolumbar region.

For training data, we used 1021 full-length X-ray images of the spines of patients with AIS taken between 2009 and 2020. The training data included supine position, supine side-bending, and wearing-brace images in addition to the standing images as our aim was to ensure that the proposed algorithm was not limited to the standing position.

During learning execution, we performed the following preprocessing steps: we resized each image to a size of 512 × 512, scaled the intensity values of each image so that the maximum and minimum values ranged from 0 to 1, and performed random black-and-white inversion processing and cropping on the input images as data augmentation.

We used the mean square error as the loss function and metric function for learning, Adam (learning factor 1.25e−4) as the optimizer, and Exponential LR (decay rate 0.96) as the scheduler. The number of learning epochs was set to 30. Our proposed method incorporates ROI identification of the thoracolumbar region into the AI algorithm for practical use in clinical practice.

### Vertebra detection

The purpose of the vertebra detection stage is to detect four corner points of each vertebral body within the ROI on the image. First, we detected > 17 candidate points of the four corners of the vertebral body for each region (upper left, upper right, lower left, and lower right). Then, we grouped each feature point by determining which vertebral body it belongs to and assigned the top 17 groups with the highest scores as the feature points of the 17 vertebral bodies.

The vertebral body to which each feature point belongs was determined by capturing the center position of the vertebral body from each point using the output of the network and grouping the feature points with those whose center positions are close to those estimated for feature points in different regions.

Since the relative vectors from each feature point to the center point of the vertebral body to which it belongs and to the center point of a different vertebral body vary greatly, it is unlikely that the detected point will be recognized as a point of a different vertebral body.

In addition, since the points in the same region of different vertebrae are located some distance from each other and the possibility of confusing points in different regions is low, we can avoid duplication of reference points by simultaneously detecting reference points in each region.

This method can directly detect the positions of reference points. In contrast, SpineNet^32^ has the disadvantage of detecting points that are clearly not on the vertebrae when it fails to detect the center point of the vertebrae. The proposed method directly detects reference points for each region, so there is little possibility that the detected result will be a point that is clearly not on a vertebra. However, a disadvantage of the proposed method is that some points may not be detected and remain missing, which requires post-processing for output.

### Learning

#### Network architecture

For feature extraction, we used Conv1–Conv5 of the pretrained model of ResNet34^31^ as the base model. The input size was 1024 × 512 × 3, and a gray-scale image of 1024 × 512 was used as the input. For each input, we used a heat map (four channels) for each feature point in the four corners of the vertebrae to identify the locations of the feature points. For one input, we simultaneously output three types of features, namely, heat map (four channels), center offset (two channels), and vertebral center offset (four channels), for each of the four vertebral angles to identify the locations of the feature points.

The loss function and metric function were defined as the sum of the loss functions of the feature point heat map, center offset, and vertebral center offset. For the loss function of the feature point heat map, we followed the variant of Focal Loss^33^ described in the SpineNet method. We used L1 Loss as the loss function for center offset and vertebral center offset. Adam (learning factor 1.25e-4) was used as the optimizer, and Exponential LR (decay rate of 0.96) was used as the scheduler. The number of learning epochs was set to 50.

### Feature point heat map

For each of the four corners of the vertebrae (upper left, upper right, lower left, and lower right), we prepared images with nonzero values only around the positions of the 17 feature points of the 17 vertebrae. They were defined using a Gaussian disk centered on the correct position of the feature points. The parameters and calculation method of the Gaussian disk are the same as those of SpineNet.

### Center offset and vertebra center offset

Center offset is used to compensate for the effects of low resolution of the output image for computational cost reduction and learning stability. It is defined as a vector field that represents the gap between the actual correct position and the position when the image is reduced to a lower resolution. Vertebral center offset is used to estimate the center position of the vertebral body from the feature points of the four corners of the vertebral body and group the feature points. It is defined as a vector that points to the relative position of the center of the vertebra from each feature point.

### CA measurement

**Figure 2:**
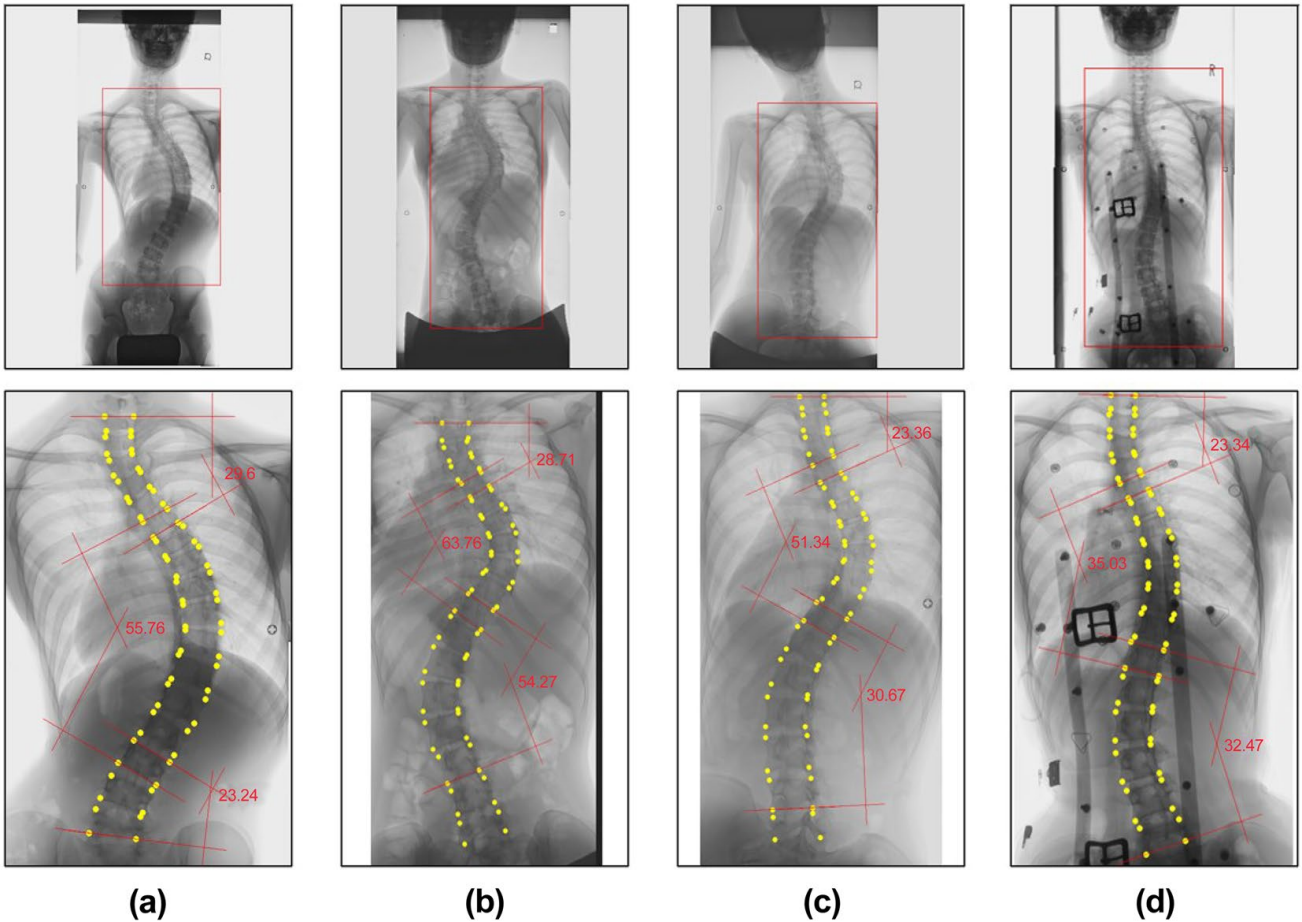
Results of CA measurement using the proposed method. The upper row shows the detected ROI indicated by a rectangle on the image, and the lower row shows the CA measurement results. Each column shows examples of (**A**) standing, (**B**) supine, (**C**) bending, and (**D**) wearing-brace X-ray images.

For each vertebra, the inclination was calculated from the points at the four corners, and the vertebra with the maximum and minimum inclination values were searched. Among the adjacent vertebrae with maxima and minima, those with tilt differences of < 5° were removed, and T1 and L5 were added to the list of vertebrae with maxima and minima. From the top, vertebrae pairs with adjacent maxima and minima were taken out and considered curves, and the difference in the inclination between vertebrae was used as the CA value for each curve. The thoracic curve maximum 2 and the lumbar curve maximum 1 were assigned to each curve in order of the highest value. Figure 2 shows examples of the proposed method applied to standing, supine, supine side-bending, and wearing-brace X-ray images.

### Evaluation

#### Dataset

The dataset was used for all postures. ROI information was used for training, and the ROIs were cropped from the original images. For data augmentation, the input images were randomly inverted in black-and-white, transformed in luminance value, and cropped by shifting the ROI position. As a part of preprocessing, the image size was resized to 1024 × 512 and then adjusted so that the values were in the range of 0 to 1, using the maximum and minimum luminance values. Standing, supine, supine side-bending, wearing-brace X-ray images taken at our hospital were used. Of these, 915 images were used for training, 106 for testing, and 155 for evaluation. Because of its clinical significance, on bending films, the CA was evaluated in the direction that corrected the curves by lateral bending.

#### Verification and analysis

Statistical analysis was performed using IBM SPSS, version 28.0 (IBM SPSS Statistics for Windows, IBM Corp., Armonk, NY, USA). ICCs according to the two-way random-effects model and two-way mixed model were used to analyze reliability; ICCs < 0.70, 0.70–0.79, 0.80–0.89, and 0.9–0.99 were considered poor, fair, good, and excellent, respectively^34^. In addition, 95% confidence intervals (CIs) were calculated.

Validation was performed using simple X-ray images of 155 cases to evaluate the interobserver reliability as the angular difference between the CA measurement by the average of six doctors and the AI measurement as well as the ICCs (Supplementary information). To assess the intraobserver reliability associated with the measurement technique, one intermediate doctor performed a second set of measurements 3 weeks after the first set using 90 radiographs to reduce the effect of memory.

## Results

### Average angle difference for all conditions

The average CA determined by the six doctors and that by the AI are shown in Table 1 for each condition. The average CA difference ranges approximately from 2.8° to 4.6°. The largest difference between the manual average CA and the AI average CA was 4.6° at the minor 2 in the bending position. Intraobserver reliability by one intermediate doctor was good, with an ICC = 0.953 for major, ICC = 0.933 for minor 1, and ICC = 0.894 for minor 2.

**Table 1:** Average Cobb angle of standing, supine, bending, and wearing-brace radiographs measured via manual measurement and the AI program.

Conditions	Number of subjects	Manual average Cobb angle (degrees)			AI average Cobb angle (degrees)			Difference between means		
		Major	Minor 1	Minor 2	Major	Minor 1	Minor 2	Major	Minor 1	Minor 2
Standing	40	39.6 ± 15.6	27.2 ± 11.7	17.7 ± 9.4	37.3 ± 15.4	24.8 ± 11.3	15.3 ± 9.9	3.2 ± 2.8	3.1 ± 2.5	2.8 ± 2.2
Supine	40	44.2 ± 10.4	31.5 ± 9.2	19.8 ± 7.0	42.5 ± 10.0	29.4 ± 10.5	17.7 ± 6.9	2.9 ± 2.2	2.8 ± 2.2	3.2 ± 3.0
Wearing brace	23	30.2 ± 13.4	20.5 ± 9.7	12.1 ± 8.2	29.5 ± 13.3	19.2 ± 9.6	10.4 ± 9.0	3.2 ± 2.6	3.0 ± 2.2	2.9 ± 2.3
Supine side-bending	52	50.6 ± 6.9	21.7 ± 8.5	9.0 ± 6.0	49.9 ± 7.5	19.9 ± 9.0	6.9 ± 8.2	3.4 ± 3.0	3.1 ± 2.9	4.6 ± 3.2

Major, Minor 1, and Minor 2 curves in order of increasing Cobb angle value. *AI* artificial intelligence.

### Evaluation by posture

The ICCs measured by the six doctors for each condition (standing, supine, wearing-brace, and supine side-bending) are shown in Table 2: ICC > 0.907 for the major, ICC > 0.882 for minor 1, and ICC > 0.878 for minor 2. The results of the AI measurements were compared with those by the six doctors for each condition, as shown in Table 3.

**Table 2:** Interobserver reliability of Cobb angle measurements made by six doctors.

Condition	Major			Minor 1			Minor 2		
	ICC (2, 1)	95% CI		ICC (2, 1)	95% CI		ICC (2, 1)	95% CI	
Standing	0.956	0.931	0.974	0.926	0.884	0.957	0.899	0.844	0.940
Supine	0.919	0.872	0.953	0.902	0.847	0.942	0.887	0.828	0.933
Wearing brace	0.951	0.915	0.975	0.882	0.797	0.940	0.889	0.816	0.942
Supine side-bending	0.907	0.844	0.952	0.893	0.817	0.945	0.878	0.700	0.970

*ICC (2, 1)* Intraclass correlation coefficient (two-way random-effects model).

**Table 3:** Interobserver reliability of Cobb angle measurements performed by six doctors and by AI.

Condition	Major			Minor 1			Minor 2		
	ICC (3, 1)	95% CI		ICC (3, 1)	95% CI		ICC (3, 1)	95% CI	
Standing	0.973	0.949	0.986	0.964	0.932	0.981	0.963	0.931	0.980
Supine	0.952	0.912	0.975	0.954	0.915	0.975	0.848	0.731	0.917
Wearing brace	0.964	0.921	0.984	0.880	0.751	0.944	0.901	0.791	0.954
Supine side-bending	0.953	0.898	0.979	0.855	0.703	0.932	0.655	0.365	0.829

*ICC (3, 1)* Intraclass correlation coefficient (two-way mixed model).

In the standing position, the ICC was > 0.96 in all groups. Especially in the major curve, the ICC exceeded 0.97, which is very high. In the supine position, the ICC was 0.952 for major, 0.954 for minor 1, and 0.848 for minor 2. In the wearing-brace condition, the ICC was 0.964 for the major, 0.880 for minor 1, and 0.901 for minor 2. In the bending condition, the ICC was 0.953 for the major, 0.855 for minor 1, and 0.655 for minor 2.

### Evaluation by angle magnitude

The errors of the six doctors and AI were evaluated for each CA size (Table 4). The error of the means were 2.23° for CA < 20°, 1.71° for 20° ≤ CA < 40°, and 2.21° for CA ≥ 40°.

**Table 4:** Evaluation of the measurement error between the AI and six doctors for measurements in the standing and supine positions.

CA	Difference between means (AI–6 doctors)	95% CI	
< 20°	− 2.23	− 3.91	− 0.56
20°–40°	− 1.71	− 2.82	− 0.59
≥ 40°	− 2.21	− 3.28	− 1.13

### Evaluation of measurements by years of experience

Table 5 shows the reliability of the measurements by years of experience and AI. The ICC was good or excellent for all groups except for the minor 2 curve in the supine side-bending position.

**Table 5:** Evaluation of measurements by years of experience.

Condition	Major			Minor 1			Minor 2		
	ICC (3, 1)	95% CI		ICC (3, 1)	95% CI		ICC (3, 1)	95% CI	
Standing	0.968	0.940	0.983	0.957	0.920	0.977	0.943	0.895	0.969
Supine	0.953	0.913	0.975	0.942	0.893	0.969	0.850	0.734	0.918
Wearing brace	0.951	0.895	0.978	0.883	0.756	0.946	0.888	0.767	0.948
Supine side-bending	0.959	0.910	0.981	0.865	0.722	0.937	0.654	0.357	0.831
Standing	0.970	0.945	0.984	0.962	0.930	0.980	0.961	0.928	0.979
Supine	0.937	0.883	0.966	0.932	0.875	0.964	0.807	0.662	0.894
Wearing brace	0.950	0.891	0.977	0.875	0.740	0.942	0.877	0.746	0.943
Supine side-bending	0.936	0.862	0.971	0.856	0.706	0.933	0.725	0.454	0.873
Standing	0.959	0.923	0.978	0.938	0.884	0.967	0.945	0.897	0.971
Supine	0.934	0.878	0.964	0.937	0.885	0.966	0.838	0.714	0.911
Wearing brace	0.967	0.927	0.985	0.846	0.686	0.928	0.878	0.747	0.943
Supine side-bending	0.928	0.846	0.967	0.815	0.629	0.912	0.578	0.252	0.786

## Discussion

### Characteristics of this study

In this study, we developed a preprocessing method for spine segmentation and vertebrae detection as well as a deep-learning architecture using CNNs to automatically measure the CA in AIS. Recently, there has been an increase in the use of machine-learning methods in various fields, and several studies have been conducted on automatic evaluation of radiological parameters of the spine. Some previous studies have used CNNs to detect spinal landmarks and measure the CA in AIS patients^18,35^. As mentioned in the previous section, this study utilized previously obtained full-length X-ray images as training data. The data collection period extended from 2009 to 2020, X-ray images taken under a wide range of imaging conditions with varying operators and imaging equipment. To enable our proposed system to handle X-ray images captured under diverse imaging conditions in clinical settings, we made two main improvements. Firstly, we employed data augmentation to enhance the variety of imaging conditions. Secondary, we structured the AI network into a two-step configuration, consisting of an ROI detection network and a vertebral body detection network. In our learning process, we utilized data augmentation techniques to generate multiple variations of a single image by adding operations, such as black-and-white inversion, left–right inversion, adding slight noise, and also performing image cropping on the input image. The learning conditions were less affected by factors, such as noise, contrast, and posture. Through the utilization of data augmentation, it became possible to construct a network that robustly identifies the ROI for the thoracolumbar spine from standing X-ray images. The robust operation of the ROI detection network resulted in an improvement in the stability of subsequent vertebra detection network. The method we used for vertebral body detection involves learning the four corner points of each vertebra and a vector that points to the center of the vertebra. This network is relatively robust against detection failures because it detects the four corner points of the vertebral body as separate heatmaps. Even if only three out of the four points are detected for a particular vertebral body, it is possible to implement post-processing to estimate the position of the fourth point based on the successfully detected three points. In comparison to the previous study, SpineNet^32^, both methods are heatmap-based vertebral detection techniques. However, while SpineNet estimates the center of the vertebral body using heatmaps, we believe that our vertebra detection method has a relatively smaller impact in cases of false detection or detection failure.

In previous reports using CNNs^15,18,19,23,36,37^ only the major curve in the standing position was evaluated. However, our study is unique because it can measure the major, minor 1, and minor 2 curves regardless of posture. Wu et al. were able to reduce the circular mean absolute error in the CA measurement to 4° by iterative training of a CNN incorporating AP and lateral views from 154 patients^18^. However, their method requires a biplanar imaging approach and may not be used when only AP image information is available. Zhang et al. developed a computer-assisted method using a deep neural network, but it still required manual intervention, such as vertebral patch assignment, and was unreliable when using in vivo radiographs to measure the CA^19^. In the previous reports, the size of the CAs was limited to less than 50°, and only two or three people measured them^15,18–20,23,34^. In addition, the highest report for ICC was > 0.93^23^, while others were below 0.90. This study supports a wide range of angle magnitudes (0°–70.5°), involves as many as six observers with different levels of experience, and can handle various imaging conditions by estimating the ROI of the thoracolumbar spine in combination with the AI. Compared with previously reported automatic measurements, the ICC was particularly high (ICC > 0.963) in the standing position, and the ICC was > 0.848 in all groups except for the minor 2 in the bending, indicating the possibility of clinical application regardless of posture.

### Evaluation by posture

In the standing, supine, wearing-brace, and supine side-bending positions, the ICCs among the evaluation by six doctors and those by AI were excellent or good, with a particularly high value of 0.973 for the major curve in the standing position. The reliability among the six doctors was excellent, with ICC for the major curve being the highest in all postures. The ICC for the major curve was also excellent in the reliability between doctors and AI. The results of the interobserver analysis suggest that AI measurements can be a good substitute for those made by doctors.

### Evaluation by angle magnitude

The difference in means between AI and doctors was not affected by the angle size, with AI tending to measure 1.7°–2.2° smaller. In the 0°–40° range, slight angle changes must be detected to optimize conservative treatment results. The error of 1.7°–2.2° appears very small because manual CA measurements are known to have errors of 3°–10°. Therefore, we believe that AI measurement is useful not only for cases with large CA that would be indicated for surgery but also for patients with a small CA that are indicated for conservative treatment or screening.

### Evaluation of measurements by years of experience

Expert, intermediate, and novice subjects all scored excellent or good, except for the minor 2 of bending. Minor 1 in the bending position, the ICCs were 0.865 and 0.856 for experts and intermediate subjects, but 0.815 for the novice subjects, suggesting that novice subjects may have been unfamiliar with the measurement in the supine side-bending. It has been reported that the experience and presence of a specialist do not affect the results of CA measurements^38^; however, this evaluation is based on the standing position only, and depending on the posture, the results may be affected. The high reliability of the AI with expert and intermediate subjects suggests that the AI can be used to accurately measure the CA regardless of the experience level of the measurer.

### Clinical applications

In this study, high accuracy was achieved not only in the standing position, but also in the side-bending position, which may allow for future reference to treatment methods. AIS has the Lenke classification^39^, and depending on the type, the range of fixation for corrective fixation can be decided^40^. Since major and minor curves are evaluated in the lateral flexion position, it may be possible in the future to determine the Lenke type using AI and determine the surgical strategy. In the present study, the accuracy was high regardless of the size of the angle; since AIS progresses gradually with growth, it is important not to miss the curve in the early stages of the disease. Therefore, the ability to detect even small angles may be useful in screening for AIS. The proposed method executes two AI networks, a ROI detection network and a vertebrae detection network. The total computation time, including execution of both AI networks, is within a few seconds per case. Quantitative benchmarking has not been performed, but this is considerably shorter than manual Cobb angle measurements. Therefore, it is expected to save time and reduce the burden on physicians in medical examinations, where a huge number of scoliosis measurements are required.

### Limitations

The limitations of this study were as follows: all of the braces used at our hospital were able to be measured successfully by our algorithm, but there were three cases in which the vertebral body could not be identified in images of braces made at other hospitals. Future studies should include a broader database of images, such as operated spines with spinal implants, infantile scoliosis, and adult scoliosis. Unlike AIS, the detection of vertebral bodies may be difficult in adult scoliosis with much spinal degeneration.

## Conclusion

The proposed method measurements showed high correlation with the doctors’ measurements regardless of the CA size, doctors’ experience, and patient posture. The proposed method showed reduced measurement error among doctors and excellent reliability assessment, indicating that it is a promising automated method for measuring CA in patients with AIS.

### Supplementary Information

Supplementary Information.

## Data Availability

The datasets used and/or analysed during the current study available from the corresponding author on reasonable request.
